# The Validity and Reliability of a Japanese Version of the Family Satisfaction in the Intensive Care Unit Survey: Multicenter Study

**DOI:** 10.31662/jmaj.2025-0403

**Published:** 2026-05-22

**Authors:** Megumi Takahashi, Satoshi Tamura, Michihiro Tsubaki, Shinya Ito, Yuichi Kataoka

**Affiliations:** 1 Department of Nursing, Kitasato University Hospital, Kitasato Minami-ku Sagamihar-shi Kanagawa-ken, Japan; 2 Department of Emergency and Critical Care Medicine, Kitasato University Hospital, Kitasato Minami-ku, Sagamihar-shi Kanagawa-ken, Japan; 3 School of Nursing, Kitasato University, Kitasato Minami-ku Sagamihar-shi Kanagawa-ken, Japan

**Keywords:** FS-ICU, critical care, intensive care unit, family research, family satisfaction, PICS-F, validity and reliability

## Abstract

**Background:**

The purpose of this study was to examine the reliability and validity of the Japanese version of the Family Satisfaction in the Intensive Care Unit (FS-ICU).

**Methods:**

In the present study, confirmatory factor analysis (CFA) was conducted to examine whether the Japanese data retained the same factor structure as reported in development studies, and CFA was performed using the maximum likelihood estimation method.

**Results:**

The CFA for the FS-ICU revealed acceptable model fit indices. The internal consistency of the FS‒ICU subscales was also evaluated. The Cronbach’s alpha for the satisfaction with care subscale was 0.967, that for the information needs subscale was 0.935, and that for the process of making decisions subscale was 0.796, indicating high reliability for the first two subscales and acceptable reliability for the third. The FS-ICU score for satisfaction with care was significantly negatively correlated with the Hospital Anxiety and Depression Scale-Anxiety (HADS-A) score and depression score, whereas information needs was significantly negatively correlated with HADS-A score (p < 0.05). However, no significant correlations between the FS-ICU and Impact of Event Scale-Revised scores were detected.

**Conclusions:**

The Japanese version of the FS-ICU questionnaire was found to have acceptable reliability and validity in a Japanese population and can be used to drive improvements in ICU care.

**Trial registration:**

Not applicable.

## Background

The admission of a patient to the intensive care unit (ICU) is often a highly stressful experience for family members ^(1)^. This is largely due to the unfamiliar and high-intensity environment of the ICU, which is characterized by advanced monitoring systems and complex medical interventions. The uncertainty surrounding the patient’s condition and the potential for poor outcomes contribute to persistent emotional strain. Moreover, family members frequently assume critical roles as caregivers and surrogate decision-makers for their loved ones, including spouses, parents, children, or siblings ^(2)^.

In this context, family satisfaction has become an important metric for evaluating the quality of ICU care ^(3)^. The most widely adopted international instrument for measuring family satisfaction in the ICU is the Family Satisfaction in the Intensive Care Unit (FS-ICU) survey ^(4)^. This survey is a self-administered questionnaire consisting of two domains: satisfaction with care (FS-ICU care) and satisfaction with decision-making (FS-ICU decision-making). Care is related to aspects such as the quality of patient care provided to the patient, the support provided to families, and the competence of healthcare professionals, and decision-making encompasses aspects such as communication frequency with medical practitioners, information consistency, and the extent of family involvement in decision-making processes. Although many ICU assessment tools focus on the perspectives of healthcare providers or patients, the FS-ICU is unique because it is focused on family satisfaction. It provides insight into two critical dimensions: the quality of treatment and the nature of interactions with medical professionals, particularly in terms of explanations and obtaining consent. Moreover, the FS-ICU includes fewer questions than many comparable tools do, thereby reducing the burden on respondents ^(3)^.

The Likert scale employed in the questionnaire is notable for its simplicity and ease of understanding.

The FS-ICU survey is the most widely utilized instrument globally for assessing family satisfaction in ICU settings. It has been translated into 23 languages and implemented in 26 countries ^(5), (6), (7), (8), (9)^. It was originally developed in Canada in 2001 and consists of 34 items. The initial validation of the FS-ICU, which was conducted across six centers, showed high internal consistency, with a Cronbach’s alpha exceeding 0.90 ^(5), (10), (11)^.

Subsequently, Wall et al. ^(4)^ refined the FS-ICU, resulting in a shortened 24-item version. This revision involved the removal of items with low response rates, poor discrimination, or conceptual redundancy. The revised scale demonstrated even higher internal consistency (Cronbach’s alpha > 0.94), and exploratory factor analysis revealed that the construct validity was supported. Additional validation studies conducted in Germany ^(6)^, France ^(12)^, and Australia ^(13)^ have further confirmed the reliability and validity of this scale.

Although the FS-ICU has been translated into Japanese ^(14)^, its psychometric properties have not yet been evaluated within the Japanese context. Thus, the reliability, validity, and applicability of this scale in Japanese clinical settings remain uncertain. Furthermore, the lack of validation limits comparability with findings from international studies.

This study aims to assess the reliability and validity of the Japanese version of the FS-ICU. Specifically, internal consistency is evaluated using Cronbach’s alpha coefficient, construct validity is assessed through confirmatory factor analysis (CFA), and criterion-related validity is also examined.

## Methods

### Data sources

In this study, a cross-sectional survey design with a self-administered questionnaire was employed. The study period is from March 2023 to March 2027. The survey was conducted in the emergency ICUs of seven tertiary hospitals in Japan. The study population consisted of family members who met all of the following criteria: the patient’s ICU stay lasted at least 48 hours, the family member was 18 years of age or older, the family member visited the patient at least once in the ICU between May 2023 and April 2024, and the family member agreed to participate in the study.

For patients who survived, questionnaires were hand-delivered or mailed to the next of kin upon discharge from the ICU or within two weeks thereafter, with a letter signed by the principal investigator enclosing a stamped, self-addressed envelope, a request to participate in the study, details of the study, and the recipient’s address. For patients who died, questionnaires were mailed to the next of kin with a letter signed by the principal investigator enclosing condolences, a request to participate in the study, details of the study, and a stamped return envelope with the recipient’s address. Satisfaction was measured using the FS-ICU.

To assess the validity of the Japanese version of the FS-ICU, the Hospital Anxiety and Depression Scale (HADS) ^(15)^ and Impact of Event Scale-Revised (IES-R) ^(16)^ were also administered via a self-administered questionnaire six months after ICU discharge. The selection of the IES-R ^(17)^ and HADS ^(18)^ was based on their extensive and well-established use in ICU family research, as well as their ability to capture key psychological constructs theoretically related to family satisfaction, including post-traumatic stress, anxiety, and depressive symptoms. This study was approved by the Ethics Committee of Kitasato University Hospital (B22-210).

### Questionnaire

#### Japanese version of the FS-ICU

The FS-ICU is used to measure satisfaction with the ICU care among patients and their family members ^(4)^. The questionnaire consists of 24 items divided into two subscales: satisfaction with care (14 items) and satisfaction with decision-making (10 items). The FS-ICU 24R questionnaire provides three summary scores.

The FS-ICU 24R is a self-administered scale, and each item is scored on a Likert scale from 1 (worst) to 5 (best). All questions are coded as integers from 1 to 5. To scale the summary scores from 0 to 100, 1 is subtracted from each item score (to obtain a range of 0 to 4) and multiplied by 25. Higher scale scores indicate greater satisfaction.

First, permission to revise this survey was requested via email from Shawna Froese, one of the FS-ICU developers. After approval was obtained, we confirmed with Japanese translators that the standard English FS-ICU questionnaire had been translated into Japanese using translation and culture adaptation guidelines. However, its reliability and validity have not been verified. Next, we obtained permission from the Japanese translator to revise the version to verify its reliability and validity.

A panel of 11 professionals in emergency and intensive care (one physician, seven nurses, and three nursing university professors) discussed the survey. Six family members completed a preliminary Japanese FS-ICU 24 survey, and their comprehension was evaluated. Regarding the change in wording, in response to the question “Question 23: Did you feel that you were in control of the care of your family member (patient)?”, the participants in the preliminary survey noted, “I don’t know what I was in control of” and “I can’t control the treatment.” There was concern that the interpretation of the words “control” and “care” would differ depending on the respondent, which would affect the answers. In response, the 11 professionals in emergency and intensive care (one doctor, seven nurses, and three nursing university professors) discussed the matter and created an alternative.

Additionally, the original author confirmed the intent of the term, and the question was revised to “Do you feel that the treatment (care) of your family member (patient) was as you wanted it?” to convey the intent of the question. Notably, some parts were difficult to understand, and a word with multiple meanings that may lead to misunderstanding were discussed and eventually replaced with the appropriate phrase without changing the meaning of the original version.

#### HADS

The HADS is a simple, validated, and widely used questionnaire to measure anxiety and depression ^(19), (20)^. The HADS ^(4)^ has been validated in many populations, including family members of ICU and hospital ward patients ^(21)^. The survey is composed of 14 questions: 7 related to depression and 7 related to anxiety. Each question is scored from 0 to 3, with a maximum of 21 points in each subscale (HADS-Anxiety and HADS-Depression). Anxiety or depression is defined as a score of ≥ 8 on the respective scale, with higher scores indicating more anxiety or depression symptoms.

#### IES-R

Many studies that have used the IES have described its usefulness for measuring psychological stress after traumatic events ^(22), (23), (24)^. The IES-R comprises 22 items (each item is scored on a Likert-type scale from 0 to 3) in three subscales (eight intrusion items, eight avoidance items, and six hyperarousal items). The Japanese-language version of the IES-R (IES-R-J) is consistent with the English version in terms of items, subscales, and the scoring method ^(16)^.

### Statistical analysis

A three-step analysis was conducted in the present study. First, to examine whether the Japanese data retained the same factor structure as reported in the development studies, a CFA was performed using the maximum likelihood estimation method. Various statistical indicators were employed to evaluate model fit. Additionally, interitem correlations were calculated to assess the relationships among the survey items.

The χ^2^ test evaluates the discrepancy between the sample covariance matrix and the model-fitted matrix, with a nonsignificant value indicating a good model fit, although it is sensitive to sample size. The comparative fit index (CFI) measures improvement in fit compared with a null model, with values above 0.90 indicating good fit. The Tucker‒Lewis index (TLI) assesses model parsimony, where higher values closer to 1 suggest a better fit. Root mean square error of approximation (RMSEA) values of 0.05 or less indicate minimal approximation error, whereas the standardized root mean square residual (SRMR) shows the average discrepancy between observed and predicted correlations, with values near 0 indicating good fit. The Akaike information criterion (AIC) and consistent AIC were used to compare model fit, with lower values indicating better models. The Bayesian information criterion (BIC) helps select models with varying parameters, preferring those with the lowest BIC values.

Second, internal consistency is assessed using Cronbach’s alpha coefficient. Finally, to examine the construct validity of the FS-ICU, correlation coefficients with the HADS and IES-R were calculated. Mplus (version 3.0) was used for CFA. Descriptive statistics and internal consistency reliability coefficients were calculated using IBM SPSS for Windows, version 25.0 (IBM SPSS Japan, Tokyo, Japan).

## Results

**Figure 1.**
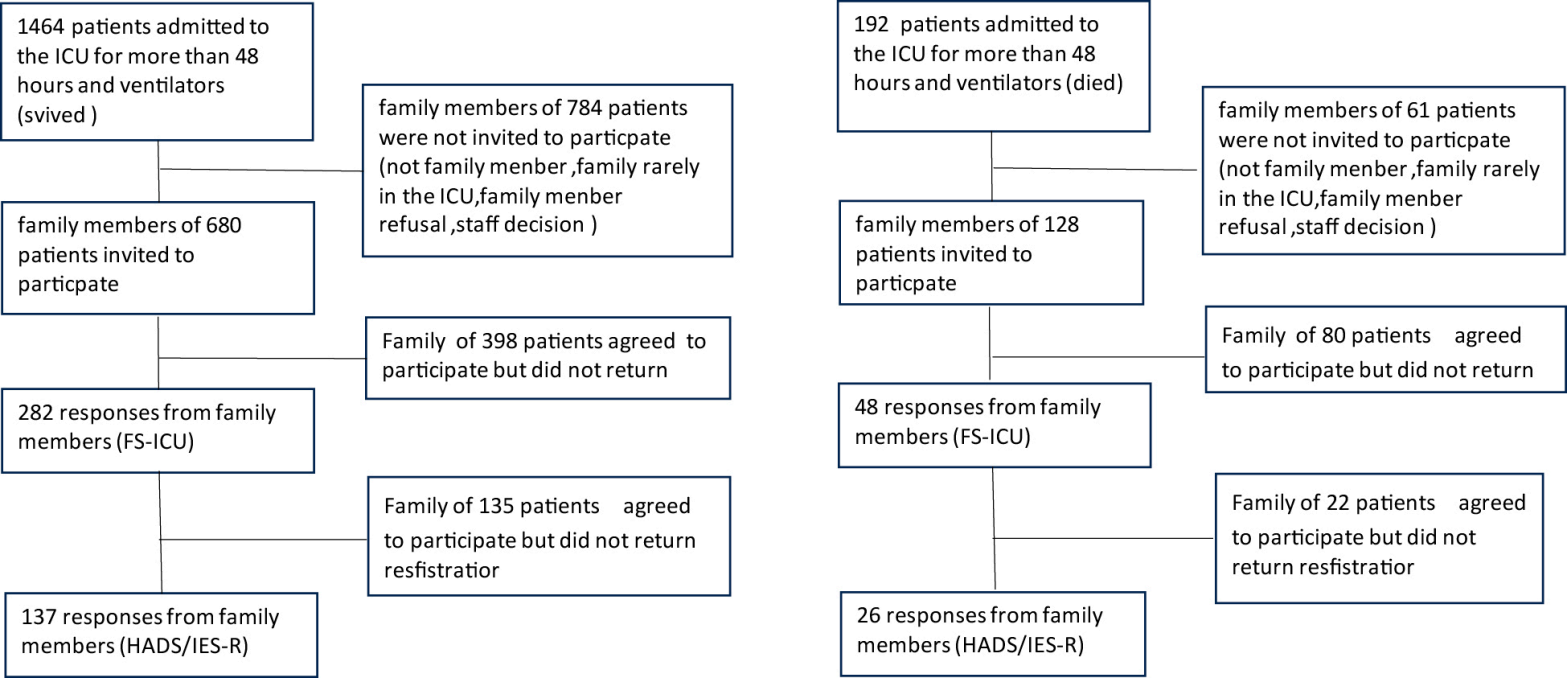
The target patient.

After this survey was conducted, responses were obtained from 330 participants. The target patients were selected as shown in Figure 1. On the basis of the FS-ICU criteria, 15 participants who left eight or more of the 26 FS-ICU items unanswered were excluded from the analysis, leaving data from 315 participants for analysis. The HADS and IES-R were administered six months after the FS-ICU survey, and 160 participants responded, of whom 151 answered all three surveys properly: the FS-ICU, HADS, and IES-R. The demographic data of the respondents and the patients involved are presented in Table 1. The study group consisted of 315 close relatives (59.7% women) of patients admitted to an ICU. The participants’ mean age was 56.5 years (standard deviation = 16.4), ranging from 23 to 89 years. A total of 46% of the relatives were spouses or partners, 16.2% were children, 14.9% were parents, 6.7% were siblings, and 16.2% were other family members. The number of patients who died during the ICU stay was 47 (14.9%).

**Table 1.** Demographics of Patients and Respondents.

		Patients				Family		
Age (n, M, SD)			58.9	27.1			56.5	16.4
Sex, (n, %)								
	Man		190	60.3%			117	37.1%
	Woman		125	39.7%			188	59.7%
	Missing		0	0.0%			10	3.2%
~~~~~~								
Reason for entry (n, %)								
	I00-I99		138	43.8%				
	J00-J99		45	14.3%				
	K00-K93		34	10.8%				
	S00-T98		25	7.9%				
	A00-B99		19	6.0%				
	OTHER		54	17.1%				
Severity score (n, M)								
	SOFA (Survived)		237	7				
	APACHE (Survived)		197	22.5				
	PIM3 (Survived)		31	1.1
	SOFA (Died)		46	10				
	APACHE (Died)		30	30.5				
	PIM3 (Died)		1	1				
ICU Stay, (M)								
	Survived		15					
	Died		12					
Outcome, (n, %)								
	Survived		268	85.1%				
	Died		47	14.9%				
Family Member, (n, %)								
	Spouse or partner						145	46.0%
	Child						51	16.2%
	Parent						47	14.9%
	Sibling						21	6.7%
	Other						51	16.2%
~~~~~~								
FS-ICU (n, M, SD)								
	Satisfaction with care					307	72.5	16.7
	Information needs					313	73.3	18.7
	Process of making decisions					312	69.4	19.5
HADS (n, M, SD)								
	Anxiety					151	6.0	4.3
	Depression					151	6.8	4.2
IES-R (n, M, SD)								
	Intrusion					153	6.2	6.1
	Avoidance					154	5.0	5.4
	Hyperarousal					153	3.7	4.1

APACHE: Acute Physiology and Chronic Health Evaluation; HADS: Hospital Anxiety and Depression Scale; ICM: intensive care medicine; IES-R: Impact of Event Scale-Revised; M: mean; PIM3: Pediatric Index of Mortality 3; SD: standard deviation; SOFA: Sequential Organ Failure Assessment.

### Confirmatory factor analysis and internal reliability

**Figure 2.**
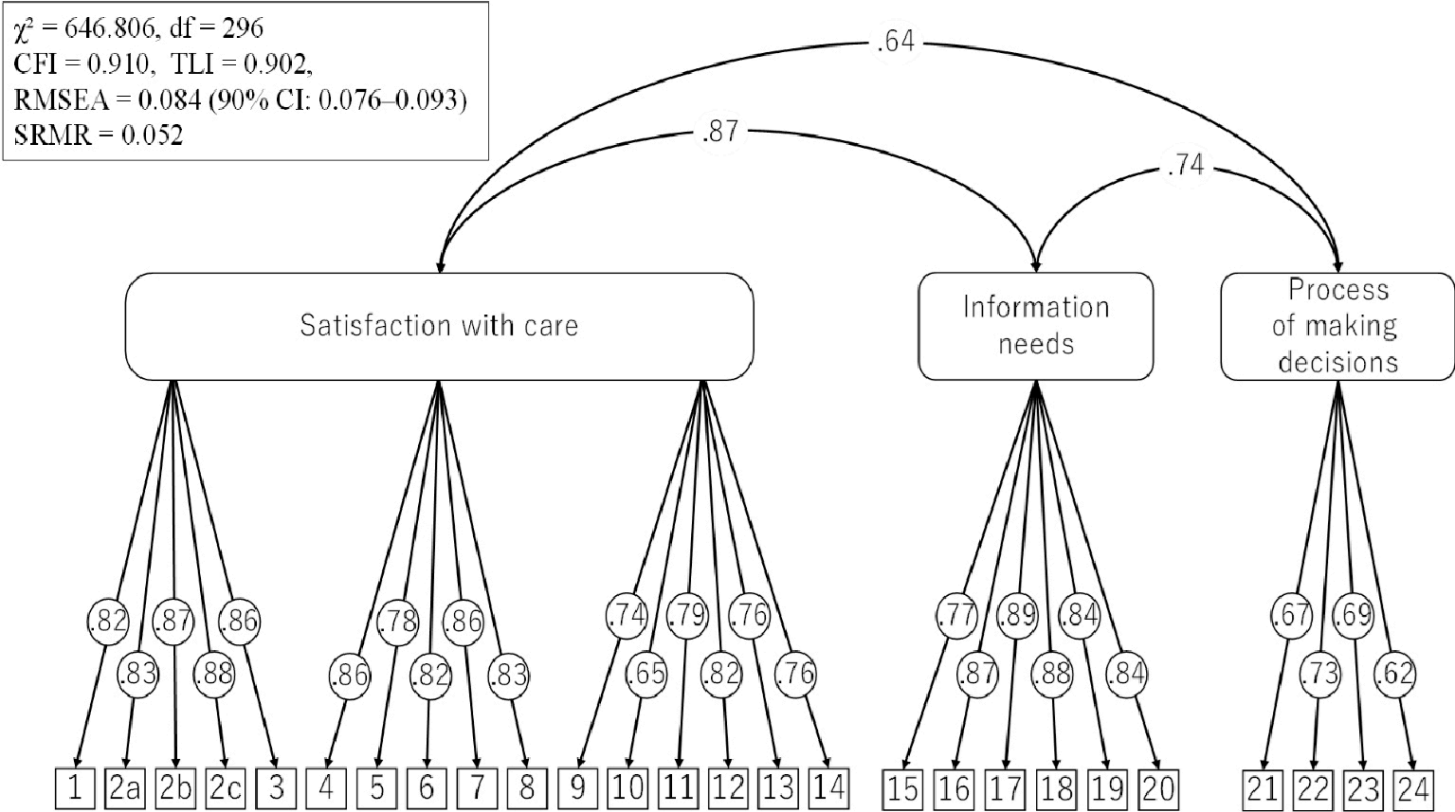
The CFA for the FS-ICU.

To conduct the CFA, response rates by item and interitem correlations of the FS-ICU were calculated (Table 2 and 3). For the CFA, the standardized estimates and the coefficients of determination (R^2^) were calculated, and the results are presented in Table 4. The CFA for the FS-ICU showed acceptable model fit indices. A three-factor model was tested, yielding the following results: χ^2^ = 646.806, degrees of freedom = 296, CFI = 0.910, TLI = 0.902, RMSEA = 0.084 (90% confidence interval: 0.076-0.093), SRMR = 0.052, AIC = 34758.9, and BIC = 34930.0. These values indicated that the model fit the data acceptably. Although the RMSEA suggested moderate fit, other indices, such as the CFI and SRMR, met conventional thresholds for good fit. The internal consistency of the FS‒ICU subscales was also evaluated. The Cronbach’s alpha for the satisfaction with care subscale was 0.967, that for the information needs subscale was 0.935, and that for the process of making decisions subscale was 0.796, indicating high reliability for the first two subscales and acceptable reliability for the third (Figure 2).

**Table 2.** Response Patterns by the FS-ICU Scores.

		M	SD	FS-ICU Score																
				100			75			50			25			0			Missing	
				n	%		n	%		n	%		n	%		n	%		n	%
1	Courtesy, respect, and compassion by staff toward patients	76.9	18.5	93	29.5		145	46.0		64	20.3		3	1.0		0	0.0		10	3.2
2a	Management of pain	73.6	19.8	78	24.8		136	43.2		79	25.1		8	2.5		0	0.0		14	4.4
2b	Management of breathlessness	72.5	19.4	66	21.0		141	44.8		81	25.7		6	1.9		1	0.3		20	6.3
2c	Management of agitation	72.8	21.3	79	25.1		129	41.0		77	24.4		11	3.5		2	0.6		17	5.4
3	How well the staff considered family needs	75.2	20.2	88	27.9		138	43.8		70	22.2		3	1.0		3	1.0		13	4.1
4	How well the staff provided emotional support toward family	71.0	20.3	66	21.0		130	41.3		93	29.5		9	2.9		1	0.3		16	5.1
5	Coordination and teamwork by staff	72.1	20.8	71	22.5		135	42.9		77	24.4		13	4.1		1	0.3		18	5.7
6	Courtesy, respect, and compassion by staff toward family	76.3	19.4	94	29.8		138	43.8		69	21.9		3	1.0		1	0.3		10	3.2
7	Skill and competence of nurses	74.4	19.4	83	26.3		136	43.2		80	25.4		5	1.6		0	0.0		11	3.5
8	Communication by nurses	71.4	21.6	75	23.8		129	41.0		82	26.0		17	5.4		1	0.3		11	3.5
9	Skill and competence of doctors	78.8	21.2	126	40.0		111	35.2		60	19.0		8	2.5		1	0.3		9	2.9
10	Atmosphere of the ICU waiting room	59.2	22.3	35	11.1		77	24.4		136	43.2		34	10.8		4	1.3		29	9.2
11	Atmosphere of the ICU	67.5	20.4	58	18.4		102	32.4		130	41.3		7	2.2		1	0.3		17	5.4
12	Participation in daily rounds by the family	71.3	21.1	54	17.1		79	25.1		72	22.9		5	1.6		1	0.3		104	33.0
13	Participation in patient care by family	71.3	20.5	56	17.8		90	28.6		79	25.1		4	1.3		1	0.3		85	27.0
14	Satisfaction with the level or amount of care the patient received	77.8	21.0	115	36.5		119	37.8		62	19.7		8	2.5		1	0.3		10	3.2
15	Frequency of communication by doctors	68.7	24.0	76	24.1		108	34.3		96	30.5		21	6.7		5	1.6		9	2.9
16	Willingness of staff to answer questions	74.3	20.7	91	28.9		130	41.3		81	25.7		8	2.5		1	0.3		4	1.3
17	Staff provided understandable explanations	73.1	20.6	85	27.0		127	40.3		92	29.2		7	2.2		1	0.3		3	1.0
18	Honesty of information provided about the patient’s condition	75.7	19.2	92	29.2		136	43.2		77	24.4		3	1.0		0	0.0		7	2.2
19	Completeness of information about what was happening	75.2	21.7	104	33.0		117	37.1		76	24.1		11	3.5		1	0.3		6	1.9
20	Consistency of information about the patient’s condition	74.0	21.5	95	30.2		121	38.4		83	26.3		11	3.5		1	0.3		4	1.3
21	Feel included in the decision-making process	74.4	28.9	133	42.2		85	27.0		57	18.1		15	4.8		18	5.7		7	2.2
22	Feel supported during the decision-making process	75.3	21.6	91	28.9		152	48.3		54	17.1		6	1.9		7	2.2		5	1.6
23	Feel control over the care of the patient	78.2	23.2	132	41.9		105	33.3		59	18.7		9	2.9		5	1.6		5	1.6
24	Adequate time to address concerns and answer questions	49.2	24.5	21	6.7		57	18.1		143	45.4		63	20.0		23	7.3		8	2.5

FS: family satisfaction; ICU: intensive care medicine; M: mean; SD: standard deviation.

**Table 3.** Matrix of Correlation Coefficients between FS-ICU Items.

		1	2a	2b	2c	3	4	5	6	7	8	9	10	11	12	13	14	15	16	17	18	19	20	21	22	23	24
1	Courtesy, respect, and compassion by staff toward patients	-																									
2a	Management of pain	0.79	-																								
2b	Management of breathlessness	0.75	0.80	-																							
2c	Management of agitation	0.77	0.75	0.80	-																						
3	How well the staff considered family needs	0.71	0.67	0.65	0.72																				
4	How well the staff provided emotional support toward family	0.71	0.70	0.67	0.75	0.80	-																				
5	Coordination and teamwork by staff	0.68	0.67	0.68	0.71	0.66	0.71	-																			
6	Courtesy, respect, and compassion by staff toward family	0.71	0.71	0.67	0.73	0.76	0.78	0.69	-																		
7	Skill and competence of nurses	0.70	0.69	0.74	0.76	0.69	0.69	0.69	0.72	-																	
8	Communication by nurses	0.64	0.65	0.67	0.71	0.68	0.69	0.62	0.64	0.73	-																
9	Skill and competence of doctors	0.56	0.62	0.58	0.58	0.59	0.56	0.58	0.60	0.59	0.54	-															
10	Atmosphere of the ICU waiting room	0.53	0.53	0.53	0.56	0.53	0.56	0.53	0.51	0.53	0.56	0.50	-														
11	Atmosphere of the ICU	0.67	0.66	0.67	0.64	0.59	0.63	0.62	0.60	0.60	0.60	0.54	0.64	-													
12	Participation in daily rounds by the family	0.62	0.64	0.63	0.63	0.64	0.66	0.52	0.61	0.67	0.67	0.53	0.64	0.70	-												
13	Participation in patient care by family	0.58	0.57	0.56	0.57	0.57	0.59	0.49	0.56	0.59	0.57	0.50	0.59	0.66	0.82	-											
14	Satisfaction with the level or amount of care the patient received	0.58	0.59	0.59	0.56	0.60	0.62	0.51	0.62	0.58	0.56	0.65	0.52	0.61	0.67	0.66	-										
15	Frequency of communication by doctors	0.55	0.52	0.54	0.60	0.58	0.56	0.44	0.49	0.53	0.61	0.57	0.55	0.60	0.61	0.58	0.56	-									
16	Willingness of staff to answer questions	0.59	0.56	0.55	0.59	0.71	0.69	0.51	0.68	0.61	0.65	0.54	0.53	0.57	0.67	0.63	0.60	0.67	-								
17	Staff provided understandable explanations	0.65	0.63	0.60	0.63	0.69	0.70	0.56	0.65	0.67	0.68	0.53	0.54	0.63	0.68	0.60	0.62	0.64	0.81	-							
18	Honesty of information provided about the patient’s condition	0.62	0.58	0.61	0.62	0.65	0.61	0.54	0.60	0.62	0.62	0.58	0.55	0.62	0.67	0.62	0.65	0.66	0.73	0.80	-						
19	Completeness of information about what was happening	0.60	0.55	0.61	0.62	0.65	0.62	0.49	0.57	0.61	0.60	0.54	0.51	0.54	0.63	0.53	0.57	0.61	0.67	0.71	0.78	-					
20	Consistency of information about the patient’s condition	0.59	0.58	0.60	0.65	0.68	0.66	0.56	0.62	0.59	0.66	0.60	0.55	0.59	0.68	0.56	0.64	0.65	0.67	0.72	0.78	0.75	-				
21	Feel included in the decision-making process	0.24	0.26	0.32	0.31	0.31	0.32	0.15	0.23	0.29	0.29	0.22	0.34	0.24	0.36	0.25	0.30	0.39	0.34	0.35	0.33	0.34	0.33	-			
22	Feel supported during the decision-making process	0.39	0.40	0.42	0.45	0.44	0.47	0.35	0.40	0.37	0.43	0.41	0.43	0.36	0.41	0.35	0.43	0.54	0.49	0.47	0.46	0.48	0.50	0.63	-		
23	Feel control over the care of the patient	0.40	0.41	0.42	0.47	0.42	0.42	0.31	0.36	0.38	0.36	0.39	0.40	0.36	0.43	0.36	0.47	0.44	0.39	0.44	0.47	0.47	0.49	0.53	0.52	-	
24	Adequate time to address concerns and answer questions	0.41	0.41	0.46	0.44	0.44	0.42	0.34	0.34	0.39	0.40	0.31	0.40	0.40	0.48	0.37	0.34	0.46	0.44	0.47	0.43	0.42	0.45	0.47	0.43	0.42	-

FS: family satisfaction; ICU: intensive care unit; M: mean; SD: standard deviation.

**Table 4.** Standardized Estimates from the Confirmatory Factor Analysis of FS-ICU.

			F1	F2	F3	R^2^
Satisfaction with care (Cronbach’s alpha = 0.967)						
	1	Courtesy, respect, and compassion by staff toward patient	0.82			0.67
	2a	Management of pain	0.83			0.69
	2b	Management of breathlessness	0.87			0.75
	2c	Management of agitation	0.88			0.77
	3	How well staff considered family needs	0.86			0.74
	4	How well the staff provided emotional support toward family	0.86			0.74
	5	Coordination and teamwork by staff	0.78			0.60
	6	Courtesy, respect, and compassion by staff toward family	0.82			0.67
	7	Skill and competence of nurses	0.86			0.74
	8	Communication by nurses	0.83			0.69
	9	Skill and competence of doctors	0.74			0.55
	10	Atmosphere of the ICU waiting room	0.65			0.42
	11	Atmosphere of the ICU	0.79			0.62
	12	Participation in daily rounds by the family	0.82			0.67
	13	Participation in patient care by family	0.76			0.58
	14	Satisfaction with the level or amount of care the patient received	0.76			0.58
Information needs (Cronbach’s alpha = .935)						
	15	Frequency of communication by doctors		0.77		0.59
	16	Willingness of staff to answer questions		0.87		0.75
	17	Staff provided understandable explanations		0.89		0.80
	18	Honesty of information provided about the patient’s condition		0.88		0.77
	19	Completeness of information about what was happening		0.84		0.71
	20	Consistency of information about the patient’s condition		0.84		0.71
Process of making decisions (Cronbach’s alpha = 0.796)						
	21	Feel included in the decision-making process			0.67	0.45
	22	Feel supported during the decision-making process			0.73	0.54
	23	Feel control over the care of the patient			0.69	0.47
	24	Adequate time to address concerns and answer questions			0.62	0.38

ICU: intensive care unit.

### Construct validity

To examine the criterion-related validity of the FS-ICU, correlation coefficients between the FS-ICU, HADS, and IES-R scale scores were calculated (Table 5). The FS-ICU score for satisfaction with care was significantly negatively correlated with HADS-Anxiety score and HADS-Depression score, whereas information needs was significantly negatively correlated with HADS-Anxiety score (*p* < 0.05). However, no significant correlations were detected between the FS-ICU and IES-R scale scores. The observed negative correlations between the FS-ICU subscales and HADS scores suggest that higher family satisfaction with ICU care is associated with lower levels of anxiety and depressive symptoms among family members. These findings support the convergent validity of the Japanese version of the FS-ICU, as psychological distress has been theoretically and empirically linked to family experiences in the ICU.

**Table 5.** Correlation Coefficient Among Scale Scores.

		1		2		3		4		5		6		7		8	
1	Satisfaction with care (FS-ICU)	-															
2	Information needs (FS-ICU)	0.84	^**^	-													
3	Process of making decisions (FS-ICU)	0.57	^**^	0.62	^**^	-											
4	Anxiety (HADS)	-0.19	^*^	-0.17	^*^	-0.14		-									
5	Depression (HADS)	-0.17	^*^	-0.14		-0.14		0.70	^**^	-							
6	Intrusion (IES-R)	-0.11		-0.07		-0.07		0.62	^**^	0.49	^**^	-					
7	Avoidance (IES-R)	-0.15		-0.10		-0.02		0.54	^**^	0.45	^**^	0.78	^**^	-			
8	Hyperarousal (IES-R)	-0.07		-0.01		-0.05		0.65	^**^	0.54	^**^	0.78	^**^	0.68	^**^	-	

FS: family satisfaction; HADS: Hospital Anxiety and Depression Scale; ICU: intensive care unit; IES-R: Impact of Event Scale-Revised. ^*^p < 0.05. ^**^p < 0.01.

## Discussion

This study aimed to evaluate the reliability and validity of the Japanese version of the FS-ICU-24. During the questionnaire development process, face and content validity were addressed through expert review and preliminary testing. Structural validity was supported statistically via CFA, and criterion-related validity was demonstrated by significant correlations with the HADS. In contrast, the lack of significant correlations between the FS-ICU and IES-R scores may reflect differences in the psychological constructs assessed by these instruments, as family satisfaction with ICU care is more closely related to emotional distress, such as anxiety and depression, than to longer-term trauma-related symptoms.

Reliability was assessed using Cronbach’s alpha coefficients, which indicated good to acceptable internal consistency across all the subscales. Although the three-factor structure identified in this study differs from the original two-factor structure reported in the initial development study, it is consistent with findings from validation studies conducted in the United Kingdom ^(25)^, Thailand ^(26)^, and Chile ^(27)^, suggesting cross-cultural variability in the instrument’s factor structure.

Taken together, these findings support the reliability and validity of the Japanese version of the FS-ICU-24. The instrument shows strong potential as a tool for assessing family satisfaction with ICU care in Japanese clinical settings.

The three-factor structure identified in this study comprises “satisfaction with care,” “satisfaction with information,” and “satisfaction with the decision-making process.” This differs from the original two-factor structure of the FS-ICU (care and decision-making), which suggests that in the Japanese context, decision-making may be perceived as two distinct components: information provision and decision-making support. This divergence may reflect cultural, institutional, and linguistic factors that are specific to the Japanese healthcare system.

In Japan, visitation in the ICU is often restricted because of infection control protocols ^(28)^. During the study period, visitation restrictions related to the coronavirus disease 2019 (COVID-19) pandemic were still partially in place in many Japanese ICUs, which may have reduced opportunities for family members to directly observe or engage in aspects of care. Moreover, traditionally restrictive visitation policies and structural characteristics of Japanese ICUs may have further constrained family participation in care and decision-making. Furthermore, structured family conferences aimed at shared decision-making are still in the early stages of implementation, and opportunities for patients and families to actively participate in care decisions remain limited. Unlike in some Western countries, where electronic medical records are more accessible to patients and families, information in Japan is predominantly delivered face-to-face ^(29)^. As a result, the nature and quality of communication between healthcare professionals and family members can have a substantial effect on how decisions are perceived and made ^(30)^.

Family members, often required to absorb complex medical information and make high-stakes decisions within a limited timeframe, may perceive the processes of receiving information and participating in decisions as psychologically distinct experiences. Even if they are satisfied with the information provided, they may not feel genuinely involved in the decision-making process because of the urgency and structure of the interaction.

Linguistic nuances further complicate this issue. For example, in item Q23, translation difficulties related to abstract terms such as “control” and “care” introduced ambiguity, potentially altering respondent interpretation. These factors likely contributed to the differentiation between information and decision-making as separate factors in the analysis.

Notably, the three-factor structure identified in this study aligns with findings from other countries. In the United Kingdom’s FREE study, the decision-making domain was similarly subdivided into “information provision” and “decision support.” Comparable structures have also been reported in Chile and Thailand. These findings suggest that perceptions of information quality and involvement in decisions may differ across cultural and healthcare contexts, shaped by language, medical practices, and the degree of family engagement in care. Thus, the three-factor model observed in the Japanese version of the FS-ICU-24 may not be unique to Japan but rather reflect the instrument’s adaptability to diverse clinical and cultural environments.

This study has several limitations. First, although exploratory and confirmatory factor analyses supported the statistical validity of the three-factor structure, its interpretability may be influenced by cultural and institutional factors, and the structure may not be generalizable to other healthcare systems. The separation of “information” and “decision-making” may reflect specific characteristics of Japanese medical culture and linguistic interpretation, necessitating caution when findings are compared with models from other countries.

Second, items 12 and 13 showed relatively high rates of missing responses, which may reflect contextual factors rather than deficiencies in the items themselves. Data collection was conducted in 2023, when visitation restrictions related to the COVID-19 pandemic were still partially in place in many Japanese ICUs. As a result, some family members may have had limited opportunities to directly observe or engage in aspects of care addressed by these items, making them difficult to answer. In addition, the traditionally restrictive visitation policies and structural characteristics of Japanese ICUs may have further limited family involvement, contributing to higher non-response rates for items related to participation and presence.

Third, though face and content validity were addressed through expert review and preliminary testing, these assessments were not conducted using standardized methods. For example, item Q23 was revised due to concerns about ambiguous language identified during pretesting, highlighting the potential impact of translation quality on validity.

Fourth, the study was conducted at only seven hospitals across Japan, raising the possibility of institutional or regional bias. Additionally, the follow-up survey conducted six months later yielded a limited response rate for the HADS and IES-R, restricting the ability to fully assess construct validity.

Future research should include a broader range of medical institutions and incorporate qualitative methods to further explore cultural adaptations of the FS-ICU-24 items.

## Conclusions

The translated Japanese version of the FS-ICU-24 questionnaire was shown to be a reliable instrument, in terms of consistency and homogeneity, for measuring family satisfaction with care in ICUs. The psychometric testing results indicate that the included items are suitable and appropriate for measuring what they intend to measure, that is, relatives’ satisfaction with care and decision-making in the ICU. The internal structure of the questionnaire revealed a pattern that differed from the results reported by Wall et al. ^(4)^. In their study, two subscales concerning satisfaction with care and satisfaction with decision-making emerged.

However, the factor analysis in this study might indicate that satisfaction with care provided to the patient and satisfaction with care directed toward the family member constitute two different subscales.

Research on FS-ICU and the safeguarding of family members in an ICU on the basis of traditions, culture, and knowledge needs to be conducted.

## Article Information

### Acknowledgments

We would like to express our sincere gratitude to the research staff at all participating institutions for their invaluable contributions to data collection and study implementation. In particular, we wish to acknowledge the following individuals for their dedicated support and commitment:

Kochi Health Sciences Hospital

·Shiho Okabayashi, Certified Nurse Specialist in Critical Care Nursing

SHOWA University Fujigaoka Hospital

·Mayumi Kumazawa, Nurse Supervisor

Nagoya University Hospital

•Naoki Ichikawa, Nurse Supervisor

•Daisuke Kasugai, MD

Nippon Medical School Hospital

·Masatomo Suzuki, Certified Nurse Specialist in Family Nursing

Seirei Hamamatsu General Hospital

•Takahiro Atsumi, Center Director

•Tomoko Kato, Certified Nurse Specialist in Family Nursing

Aichi Children’s Health and Medical Center

•Sho Wada, MD

Kitasato University Hospital

•Eri Mineo, MD

•Shigeyuki Naito, Head Nurse

•Yuni Kamimura, Nurse Supervisor

•Shota Numanoi, RN

•Azuma Kimishima, Nurse Supervisor

•Shuichi Matsuda, Nurse Supervisor

•Kazuma Seki, RN

Their dedicated efforts and support were instrumental to the successful completion of this study, and we deeply appreciate their valuable contributions.

### Author Contributions

Megumi Takahashi and Satoshi Tamura contributed to the study conception, design, acquisition of data, and analysis and interpretation of the data; drafted the article; and approved the final version to be published. Michihiro Tsubaki contributed to the analysis and interpretation of the data, drafted the article, and approved the final version to be published. Shinya Ito and Yuichi Kataoka contributed to the analysis and interpretation of the data, revised the manuscript critically for important intellectual content, and approved the final version to be published.

### Conflicts of Interest

None

### IRB Approval Code and Name of the Institution

The Institutional Review Board of Kitasato University Hospital approved the study protocol (B22-210).

### Ethics Approval and Consent to Participate

This study was approved by the Ethics Committee of Kitasato University Hospital (approval No. B22-210) and conducted in accordance with the Declaration of Helsinki. Written information about the study was provided to family members of ICU patients, and informed consent was considered obtained when they voluntarily completed and returned the questionnaire.

### Availability of Data and Materials

The datasets generated and analyzed during the current study are not publicly available owing to participant privacy considerations, but are available from the corresponding author upon reasonable request.
